# Buffy Coat Transcriptomic Analysis Reveals Alterations in Host Cell Protein Synthesis and Cell Cycle in Severe COVID-19 Patients

**DOI:** 10.3390/ijms232113588

**Published:** 2022-11-05

**Authors:** Liliane Tavares de Faria Cavalcante, Guilherme Cordenonsi da Fonseca, Luciane Almeida Amado Leon, Andreza Lemos Salvio, Otávio José Brustolini, Alexandra Lehmkuhl Gerber, Ana Paula de Campos Guimarães, Carla Augusta Barreto Marques, Renan Amphilophio Fernandes, Carlos Henrique Ferreira Ramos Filho, Rafael Lopes Kader, Marisa Pimentel Amaro, João Paulo da Costa Gonçalves, Soniza Vieira Alves-Leon, Ana Tereza Ribeiro Vasconcelos

**Affiliations:** 1 Laboratório de Bioinformática, Laboratório Nacional de Computação Científica, Petrópolis, Rio de Janeiro 25651-076, Brazil; 2 Laboratório de Desenvolvimento Tecnológico em Virologia, Instituto Oswaldo Cruz/FIOCRUZ, Rio de Janeiro 21040-360, Brazil; 3 Laboratório de Neurociências Translacional, Universidade Federal do Estado do Rio de Janeiro, Rio de Janeiro 20211-040, Brazil; 4 Hospital Universitário Clementino Fraga Filho, Universidade Federal do Rio de Janeiro, Rio de Janeiro 21941-617, Brazil; 5 Laboratory of Translacional Neurosciences, Biomedical Institute, Universidade Estadual do Rio de Janeiro, Rio de Janeiro 20550-013, Brazil; 6 Yale New Haven Hospital, New Haven, CT 06510, USA

**Keywords:** COVID-19, SARS-CoV-2, RNA-seq, lncRNA, biomarkers, buffy coat, FBL, ICU patients

## Abstract

Transcriptome studies have reported the dysregulation of cell cycle-related genes and the global inhibition of host mRNA translation in COVID-19 cases. However, the key genes and cellular mechanisms that are most affected by the severe outcome of this disease remain unclear. For this work, the RNA-seq approach was used to study the differential expression in buffy coat cells of two groups of people infected with SARS-CoV-2: (a) Mild, with mild symptoms; and (b) SARS (Severe Acute Respiratory Syndrome), who were admitted to the intensive care unit with the severe COVID-19 outcome. Transcriptomic analysis revealed 1009 up-regulated and 501 down-regulated genes in the SARS group, with 10% of both being composed of long non-coding RNA. Ribosome and cell cycle pathways were enriched among down-regulated genes. The most connected proteins among the differentially expressed genes involved transport dysregulation, proteasome degradation, interferon response, cytokinesis failure, and host translation inhibition. Furthermore, interactome analysis showed Fibrillarin to be one of the key genes affected by SARS-CoV-2. This protein interacts directly with the N protein and long non-coding RNAs affecting transcription, translation, and ribosomal processes. This work reveals a group of dysregulated processes, including translation and cell cycle, as key pathways altered in severe COVID-19 outcomes.

## 1. Introduction

Since the beginning of the severe acute respiratory syndrome coronavirus 2 (SARS-CoV-2) pandemic, declared on 11 March 2020 by the World Health Organization (WHO), a worldwide race has begun to establish the best diagnostic methods, to sequence variants, to develop vaccines, to discover the molecular basis of the virus pathogenesis, and to describe the gene expression profiles under different coronavirus disease 19 (COVID-19) outcomes. Patients with severe COVID-19 illness may become critically ill through developing acute respiratory distress syndrome (ARDS) [1]. A meta-analysis study has reported that the severe prognosis of the disease was noted in about 23% of COVID-19 patients, with a mortality rate of about 6% [2]. 

Several factors are related to different COVID-19 outcomes. For example, SARS-CoV-2 viral loads may be higher in severe COVID-19 than in asymptomatic and mild infections [3]. Recent studies have also related serum biomarkers to COVID-19 severity and mortality, including neurofilament light chain (NfL), glial fibrillary acidic protein (GFAp), total TAU (Microtubule-Associated Protein Tau, MAPT), and Ubiquitin carboxy-terminal hydrolase L1 (UCH-L1) [4]. These blood biomarkers have been described as important prognostic factors related to poor outcomes. Moreover, higher NfL and TAU levels have been significantly associated with the predicted risk of death [5,6]. 

RNA-seq approaches can help to understand the whole cell response scenario regarding SARS-CoV-2. A study has investigated the main differences between the different outcomes found in COVID-19 disease versus healthy controls in leukocytes and peripheral blood mononuclear cell (PBMC) samples [7,8], where the main dysregulated gene ontology (GO) terms identified were Neutrophil degranulation, Vessel damage, Platelet activation and degranulation, blood coagulation, complement activation, lymphocyte mediated immunity, and mRNA processing, among others. Buffy coat from critically ill patients admitted to the intensive care unit (ICU) positive and negative for SARS-CoV-2 showed distinctions in the expression of genes associated with protein translation, protein synthesis, SUMOylation, and ubiquitination, which were all decreased in COVID-19 positive individuals. On the other hand, tumor necrosis factor (TNF) and interleukin 6 (IL6) signaling and interferon (IFN)-mediated gene transcription-related genes were found to be up-regulated in this same group [9].

Protein–protein interaction (PPI) studies have identified SARS-CoV-2 proteins that can interact with specific host genes, disrupting cellular functions such as splicing, translation, and trafficking in order to suppress essential biological pathways such as the interferon response to viral infection [10]. SARS-CoV-2 open reading frame 6 (Orf6) interacts with nucleopore proteins (Ribonucleic Acid Export 1, RAE1; Exportin 1, XPO1; RAN Binding Protein 2, RANBP2; and nucleoporins) and causes high cytotoxicity when over-expressed [11]. SARS-CoV-2 non-structural protein 16 (NSP16) binds to specific mRNA recognition domains and acts through suppression of mRNA splicing. NSP1 binds to a precise region on the 18S ribosomal RNA in the mRNA entry channel, leading to the overall limitation of mRNA translation. Non-structural protein 8 (NSP8) and non-structural protein 9 (NSP9) bind to a component of the signal recognition particle (SRP) and inhibit membrane and secretory protein trafficking [10]. These are just a few examples of how SARS-CoV-2 can interfere with cell biology; however, as an emerging pathogen, our understanding of how it evades the immune system and the functional roles of its genes is still limited.

By inhibiting key host proteins, SARS-CoV-2, like other viruses, can modify cellular processes to avoid antiviral pathways, as well as improving the transcription and translation of its own proteins, allowing for the disease’s progression. The aim of this work was to distinguish the cellular expression profile from the aggressive and mild types of SARS-CoV-2 infection. The distinction between healthy donors and critically ill patients would reveal not only the main altered mechanisms involved in severe pathogenesis but also mechanisms modified by the SARS-CoV-2 infection irrespective of disease severity. By comparing directly with the mild group, we aimed to analyze the genes altered in the severe outcome of the disease. Understanding the altered cellular mechanisms in the severe outcome of COVID-19 can help to develop preventive policies, treatments, and drugs targeting specific pathways for this clinical condition.

## 2. Results

### 2.1. Cohort Data

A RNA-seq data set for 17 COVID-19 patients was generated, including 5 patients with mild symptoms and 12 hospitalized patients with severe symptoms. This cohort covered an age range from 46 to 72 years, and the ratio between female and male patients was 9:8. Between the mild and severe groups, no significant difference was noted in the gender composition and mean age (53 ± 6.3 and 64.5 ± 7.04, respectively). We observed a significant difference between viral loads in the hospitalized group versus the mild group (*p* = 0.0059; Table 1 and Table S1). Regarding the severity biomarkers investigated, only GFAP levels presented no statistical significance when both groups were compared. UCH-L1 (*p* = 0.0133), NfL (*p* = 0.0275), and TAU (*p* = 0.0133) levels were all found to be statistically significantly higher among hospitalized patients, when compared to mild cases.

### 2.2. Differentially Expressed Genes (DEGs) and Enrichment Analysis 

**Figure 1:**
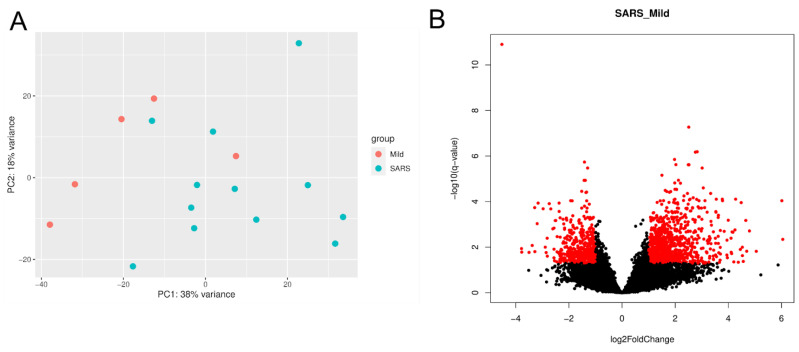
Differential expression gene analysis. The principal component analysis (**A**) shows that the RNA-seq libraries from the mild and SARS patients were grouped distinctly, except for one individual in each group. Volcano plot (**B**) displaying (with red dots) the differentially expressed genes (log_2_ fold-change > |1| and adjusted *p*-value < 0.05).

The gene expression profiles in the buffy coat of each patient with severe and mild COVID-19 were analyzed. Principal component analysis (PCA) clearly distinguished between SARS and mild groups in the first principal component (Figure 1A). When comparing SARS over mild COVID patients, among the 7813 expressed genes identified by RNA-seq, we found 1009 up-regulated genes and 501 down-regulated genes (Figure 1B).

**Figure 2:**
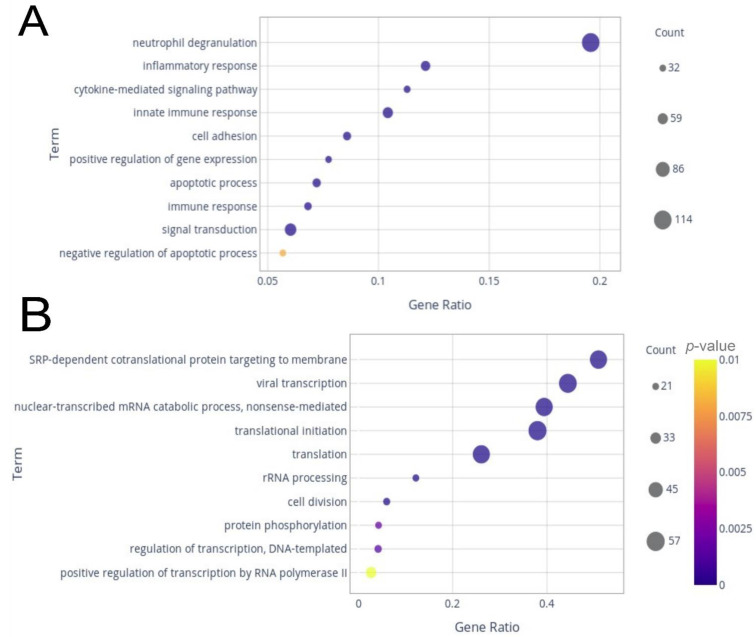
Dot plots from gene ontology biological process enrichment analysis. The plots display the top 10 terms with higher gene counts among up-regulated genes (**A**) and down-regulated genes (**B**).

Of those genes, around 10% were composed of lncRNAs, of which 133 were up-regulated and 19 were down-regulated. GO analysis for biological processes was conducted to determine the main sets of DEGs. Considering the up-regulated genes, the most prominent pathways identified were neutrophil degranulation, with more than 15% of its genes differentially expressed (Figure 2A). Additionally, inflammatory response, cytokine-mediated signaling pathway, innate immune response, cell adhesion, positive regulation of gene expression, apoptotic process, immune response, signal transduction, and negative regulation of apoptotic process were among the most enriched GO terms (Figure 2A). Half of the 10 most enriched down-regulated processes had more than 25% (gene ratio > 0.25) of genes assigned to each set as down-regulated (Figure 2B).

**Figure 3:**
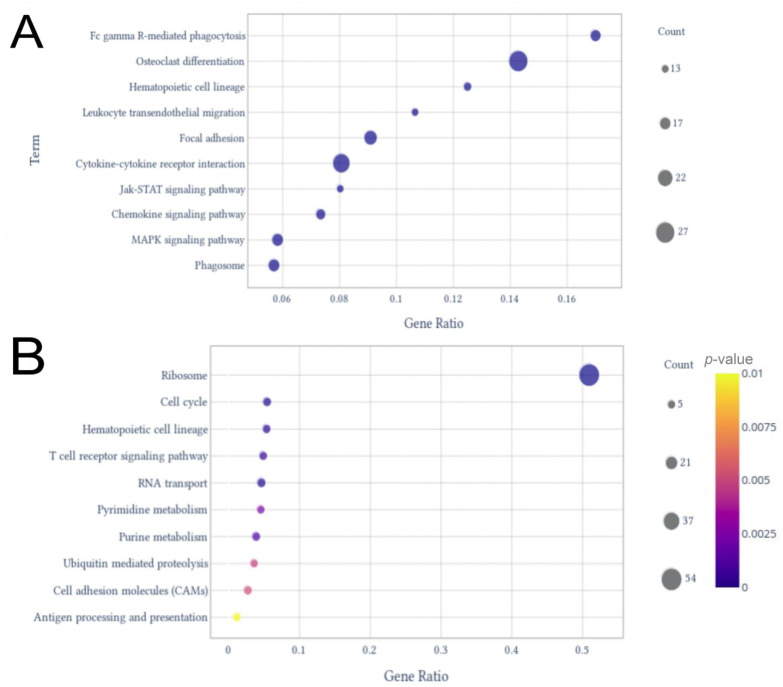
Dot plots from KEGG pathway enrichment analysis. The plots display the top 10 terms with higher gene counts among up-regulated genes (**A**) and down-regulated genes (**B**).

These included SRP-dependent co-translational protein targeting to membrane, viral transcription, nuclear-transcribed mRNA catabolic process—non-sense-mediated, translation initiation, and translation. rRNA processing, cell division, protein phosphorylation, regulation of transcription, and DNA-templated and positive regulation of transcription by RNA polymerase II completed the top 10 enriched terms by gene ratio (Figure 2B). In the KEGG analysis for the up-regulated genes, the pathways with the highest gene ratios were osteoclast differentiation and Fc gamma R-mediated phagocytosis (Figure 3A). Hematopoietic cell lineage, leukocyte trans-endothelial migration, focal adhesion, cytokine–cytokine receptor interaction, Jak-STAT signaling pathway, chemokine signaling pathway, Mitogen-Activated Protein Kinase (MAPK) signaling pathway, and phagosome were in the top 10 enriched pathways with the highest gene ratios. For the down-regulated genes, ribosome pathway and cell cycle had the highest gene ratios, followed by hematopoietic cell lineage, T cell receptor signaling pathway, RNA transport, pyrimidine metabolism, purine metabolism, ubiquitin-mediated proteolysis, cell adhesion molecules, and antigen processing and presentation (Figure 3B).

### 2.3. Interactome

**Figure 4:**
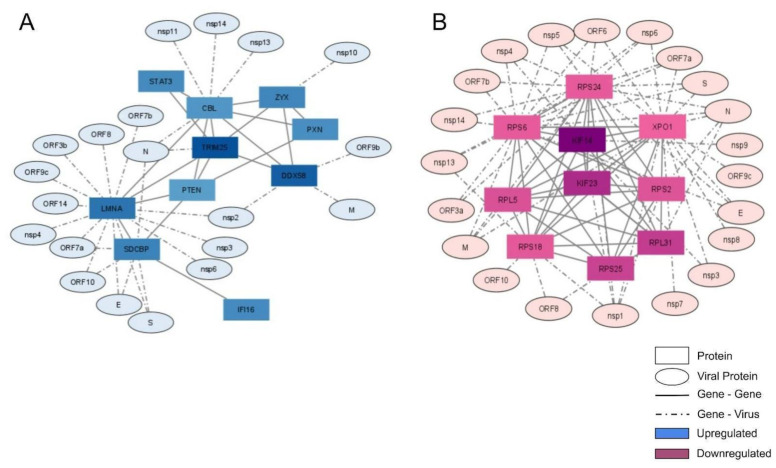
Interaction network of the 10 most connected up- and down-regulated genes and the SARS-CoV-2 proteins that interact with them. The most-connected (by edge count) of the up-regulated genes (**A**) and down-regulated genes (**B**) were used to construct an interaction network using the Interactome tool. SARS-CoV-2 proteins were added using biomedical interaction repository (biogrid) information.

We selected the 10 most connected genes in up- and down-regulated groups and plotted them in an interaction network. To verify the possible association of these genes with SARS-CoV-2 proteins, data from the SARS-CoV-2 interactome were used to link them (Figure 4).

In the up-regulated gene network, CBL (Cbl Proto-Oncogene) was the most connected protein, having seven connections with cellular proteins and four connections with viral proteins (Figure 4A). CBL acts as an E3 ubiquitin-protein ligase transferring ubiquitin to substrates and promoting their degradation by the proteasome [12]. Furthermore, among the most-connected up-regulated genes, we observed some antiviral proteins, including STAT3 (Signal Transducer And Activator Of Transcription 3), DDX58 (Antiviral innate immune response receptor RIG-I), TRIM25, and IFI16. STAT3 is a multi-functional factor involved in numerous physiological processes, including inflammation and immunity [13]. DDX58, also known as RIG-I, is an antiviral protein involved in detecting viral dsRNA and regulating the antiviral innate immune response [14]. TRIM25 is a protein induced by interferon, which is involved in the innate immune defense against viruses by mediating K63-linked polyubiquitination of DDX58, which is required to activate the downstream signaling interferon pathway [15]. TRIM25 was well-connected in the network, with six connections to cellular proteins and one connection with the viral protein N (Figure 4A). IFI16 is a protein that detects viral DNA both in the cytoplasm and nucleus [16].

In addition to antiviral proteins, we observed some correlated cytoskeleton proteins among the most-connected up-regulated genes, including LMNA, PXN, and ZYX. LMNA (or Lamin A/C) plays important roles in nuclear assembly, chromatin organization, nuclear membrane, and telomere dynamics. PXN (Paxillin) is a cytoskeleton protein involved in cellular adhesion to the extracellular matrix, the phosphorylation of which promotes cell migration [17]. ZYX (Zyxin) is a zinc-binding phosphoprotein that concentrates on focal adhesions and along the actin cytoskeleton [18]. PTEN is a protein that functions as a tumor suppressor by negatively regulating the AKT/PKB signaling pathway, thus modulating cell cycle progression and survival [19]. SDCBP (Syndecan-Binding Protein) is a protein involved in the trafficking of trans-membrane proteins and exosome biogenesis [20]. 

Most down-regulated connected genes belonged to the structural part of the ribosome (RPS6, RPS24, RPS25, RPS18, RPL31, RPL5, and RPS2; Figure 4B and Figure S1). They were overall well-connected. RPS24, like the other ribosomal proteins, is synthesized in the cytoplasm and carried to ribosomal sub-unit assembly in the nucleolus [21]. RPS25 has a well-established role in some viral replication cycles, being essential for translation initiation through IRES of hepatitis C virus (HCV) and Human T-lymphotropic virus type-1 (HTLV-1) [22]. RPS18, RPS2, RPL31, and RPL5 have been well-studied in plants, although their functions in mammals include Ribosome structure and RNA binding. 

Other down-regulated genes comprised cytoskeleton proteins (e.g., KIF14 and KIF23) and nuclear export of some cellular proteins (e.g., XPO1). KIF14 silencing interrupts cell cycle progression and induces cytokinesis failure [23]. Its knockdown inhibits cell proliferation and induces apoptosis [24]. KIF23 (also known as MKLP1) is localized mainly in the cytoplasm and nucleus. It participates in cell mitosis, comprising the centralspindlin complex [25]. XPO1 (or CRM1) is a nuclear transporter characterized as enabling traffic of proteins, RNAs, and ribosomal sub-units [26,27]. 

### 2.4. lncRNA Interactome

**Figure 5:**
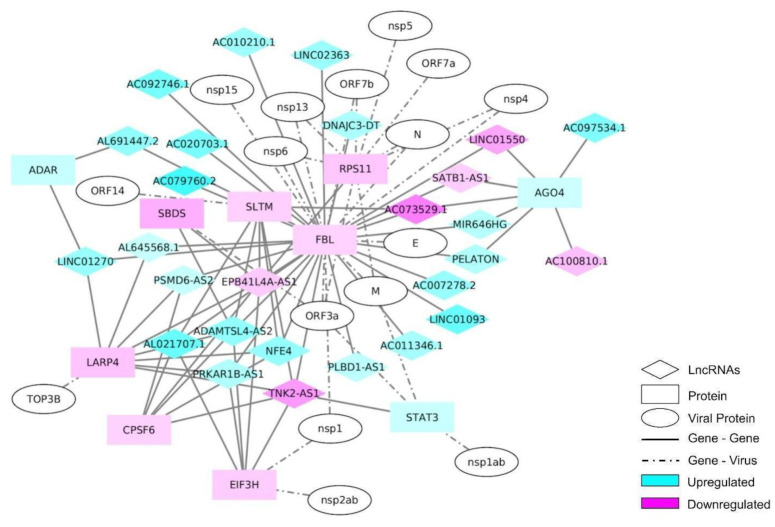
Interaction network of the most differentially expressed long non-coding RNAs (lncRNAs) and protein-coding genes, along with the SARS-CoV-2 proteins that interact with each one. An interaction network was constructed using an interactome tool with the differentially expressed lncRNAs and protein-coding genes. The SARS-CoV-2 proteins were added using biomedical interaction repository (biogrid) information.

As 10% of the DEGs identified in our RNA-seq were lncRNAs, an interactome analysis including the SARS-CoV-2 proteins was performed, in order to understand the roles of these genes during the infection. Fibrillarin (FBL) was the gene with the highest number of connections in this network, when the NEAT1 lncRNA was excluded (Figure 5). NEAT1 had multiple connections to other genes, but none of them were connected with the remainder of the network; for this reason, these connections were removed from the network (Figure S2). FBL is a 2′-O-Methyltransferase and a component of a nucleolar small nuclear ribonucleoprotein (snRNP) that participates in the first step in processing pre-ribosomal RNA [28]. The FBL was connected with the N protein of SARS-Cov-2 (Figure 5). 

As FBL presented a central role in this protein–lncRNA network, further interactome analyses were performed to understand this gene’s importance in the SARS-CoV-2 infection. These networks were constructed using the Dijkstra algorithm, in order to connect the FBL to all of the DEGs—including the expressed genes—considering the shortest distance with the highest value of log_2_ fold-change. In this way, the network connecting the FBL to all DEGs, including up- and down-regulated genes, contained 2430 genes, of which 130 were lncRNAs, 6 miscRNAs, 1 scaRNA, 4 snoRNAs, and 3 snRNAs (Table S2 and Figure S3). The network connecting the FBL to all up-regulated DEGs contained 1837 genes, including 116 lncRNAs, 5 miscRNAs, 1 scaRNA, 4 snoRNAs, and 3 snRNAs (Table S3 and Figure S4). Finally, the network connecting the FBL to all down-regulated DEGs had 1084 genes, including 21 lncRNAs and only 1 miscRNA and snoRNA (Table S4 and Figure S5). Interestingly, the ncRNAs were concentrated closer to the FBL in these networks, indicating that this gene might have an important role in the modulation of those ncRNAs during the SARS-CoV-2 infection. Regarding the genes found in those interactomes, most terms related to cell cycle were enriched in cell cycle, regulation of cell cycle, mitotic cell cycle, regulation of cell cycle process, and regulation of cell cycle phase transition (Table S5–S7). FBL is also related to ribosome functions, as it is part of the ribonucleoprotein complex that methylates rRNAs, regulating its structure and stability [29]. Ribosomal genes were found in the RNA-seq data, with the ribosome pathway being the one with the highest gene ratio in the KEGG analysis, with almost 50% of its genes down-regulated (Figure 3B). Moreover, in the interactome of the most connected down-regulated genes, 7 of 10 were ribosomal proteins (Figure 4B), and GO enrichment analysis of the FBL interactomes also identified biological processes including ribosome biogenesis, ribosome assembly, and ribosome-associated ubiquitin-dependent protein catabolic process (Tables S5–S7). Furthermore, FBL, as other ribosome biogenesis factors, is a component of stress granules [30]; these structures have been found to be impaired by SARS-CoV-2 N protein to promote viral replication [31]. 

One of the most connected up-regulated proteins in the RNA-seq interactome was Lamin A/C (LMNA), which is also a component of stress granules [32] and is highly connected with SARS-CoV-2 proteins (Figure 4A). Among all of the de-regulated genes in this work, we found several that were part of the stress granule composition (15 up-regulated genes and 19 down-regulated genes). It is interesting to note that more than 25% of these genes were in the networks constructed in Figure 4A,B and Figure 5.

The up-regulated genes AGO4, STAT3, and ADAR were also found in this network, where the latter two have already been implicated as playing a role in the COVID-19 disease [33,34]. The down-regulated genes identified in the network included SLTM, RPS11, SBDS, LARP4, CPSF6, and EIF3H (Figure 5). Two of these genes are related to ribosomes: RPS11, a component of the 40S sub-unit [35], and SBDS, which participates in ribosome maturation [36]. EIF3H and LARP4 play roles in translation regulation [37,38], while SLTM and CPSF6 are involved in the transcription process and maturation [39,40]. In total, 27 lncRNAs were identified in this network, where 21 were up-regulated and 6 were down-regulated (see Figure 5).

## 3. Discussion

There is still limited understanding of the molecular pathways altered during the SARS-CoV-2 infection [41,42]. Transcriptome analyses are valuable approaches to identify the altered genes and related pathways having many biological functions. Neutrophil de-granulation is one of the major events that modulates the immune system post-infection, as evidenced by the increased peripheral neutrophil-to-lymphocyte ratio in severe COVID-19 patients [41]. Neutrophil cells secrete granule contents, with a high capacity to cause collateral tissue damage in hypoxic environments [43]. This is a possibility of what may occur in patients with ARDS caused by SARS-CoV-2, which can lead to local and systemic hypoxia. Activation of inflammatory signaling pathways and cytokine storms are other crucial factors that lead to acute respiratory distress syndrome (ARDS) in COVID-19 patients. The excessive secretion of pro-inflammatory cytokines and chemokines leads to dysregulation of the innate immune system [44]. Interleukin IL-6 is a hallmark of the cytokine storm observed in severe COVID-19 patients [45]. STAT3 and STAT1 can activate the IL-6 amplifier to induce various pro-inflammatory cytokines and chemokines, including vascular endothelial growth factor (VEGF), monocyte chemoattractant protein-1 (MCP-1), IL-8, and IL-6 [46].

In this work, we observed higher levels of TAU total protein in the severe group than in the mild one. Indeed, it has been suggested by other authors that the SARS-CoV-2 spike S1 protein directly interacts with and binds to the TAU protein, forming an S1–TAU complex or S1–Heparin–TAU complex, which presents stronger binding, increased neurological distress, and neurodegeneration, leading to severe COVID-19 and death [4,47]. Another study has demonstrated that the up-regulation of STAT3 in microglia is related to TAU pathology and neuroinflammation. In severe COVID-19, NfL serum levels have been described as significantly higher, when compared to the overall population. Indeed, NfL levels can serve as an important prognostic risk factor for fatality in these cases [4,48]. 

Both TAU and NfL levels were higher in the severe group than in the mild one (*p* ≤ 0.05). These are two important blood biomarkers that have been widely related to severe SARS-CoV-2 infection and death [4]. This likely occurs, as these biomarkers are altered during nervous system injury due to SARS-CoV-2 presence and replication and the consequent astrocyte and glial activation, leading to cytokine storms and inflammatory responses in the CNS [4,49,50].

A virus does not carry sufficient information in its genome, thereby increasing its dependence on the host to make viral proteins. To achieve this, viral mRNAs compete with host mRNAs to access limited cellular translational resources [51]. The down-regulation of ribosomal structural genes might result from the virus–host protein interaction to suppress the translation of the host mRNA, consequently maximizing the transcription and translation of the SARS-CoV-2 genes [52]. As another consequence of this process, the host protein translation is decreased, impairing the antiviral response of the cell and also delaying the cell cycle.

**Figure 6:**
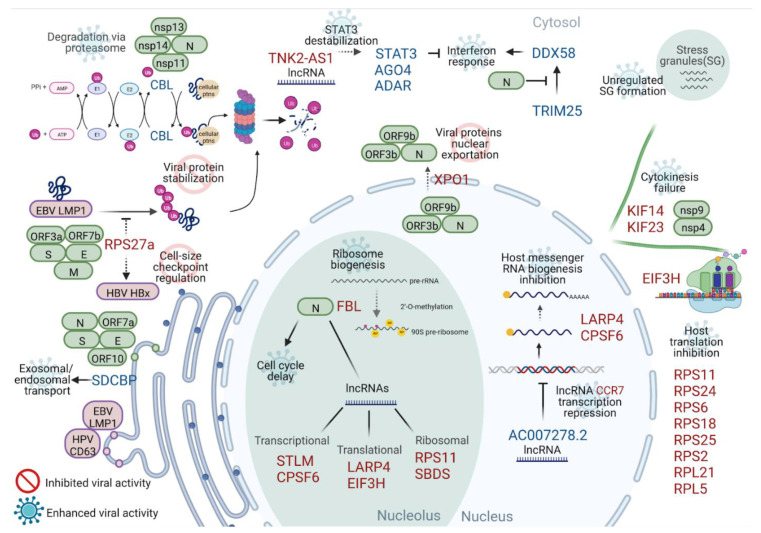
Schematic of the putative impact of DEGs on viral activity. The most-connected genes or those that participate in the main enriched pathways are placed together with SARS-CoV-2 and other virus proteins that interact with each. The host protein functions and their possible role in the cell response to the virus or inhibition due to viral infection were inferred based on the literature. Ribosome biogenesis (also through long non-coding RNA branching), cell cycle delay, and host translation inhibition are the processes more concentrated in differentially expressed genes. SARS-CoV-2 proteins are shown in green boxes; other viral proteins are shown in purple boxes. For differentially expressed genes, up-regulated genes are marked in red, while down-regulated genes are marked in blue. The pathways are shown in black, each marked with a “prohibited” icon for inhibited viral activity or a “virus” icon for enhanced viral activity. The acronym SG denotes stress granules. Created with BioRender.com.

Figure 6 highlights the main findings of this work and the biological processes already observed in the previous literature. Each proposed function is labeled according to its role: (a) in the induction of greater cellular damage caused by the viral infection (enhanced viral activity), or (b) as part of a more incisive cellular response to limit viral activity (inhibited viral activity). 

We propose that exosomal/endosomal transport may be linked to enhanced viral activity. The SDCBP protein interacts with different viral proteins, such as LMP1 from EBV and CD63 from Papillomavirus, enabling exosomal packaging and post-endocytic transport [53,54]. The up-regulation of SDCBP in the present buffy coat transcriptome, as well as the observed interaction of this protein with 5 SARS-CoV-2 viral proteins (S, E, ORF10, ORF7a, and N), might enable better virus protein traffic within the host cell (Figure 6).

Another altered function linked to cellular transport is the viral protein export mediated by XPO1. This protein plays a role in SARS-CoV-2 replication and pathogenesis by performing nuclear export of some viral proteins, including ORF3b, ORF9b, and N protein (Figure 6) [55,56,57,58]. ORF9b contributes to viral evasion from innate immunity by inhibiting type I interferon induction [59]. Therefore, its transport is very important to the viral cycle. XPO1 inhibition resulted in nuclear retention of ACE-2, diminishing its availability on the cell surface [55], and retention of key viral accessory proteins, reducing viral replication and immunopathogenesis in SARS-CoV-2 and MERS-CoV infection [60,61]. Therefore, XPO1 down-regulation would inhibit and limit viral replication (Figure 6), either by reducing the export of viral proteins from the nucleus, or by decreasing the presentation of the virus-cell receptor (ACE2) on the cell surface.

Many viruses can recruit cellular E3 ligases (e.g., CBL, E6AP, cullin-RING) to target anti-viral proteins for degradation [62]. Papillomavirus (HPV) E6 and E7 proteins [63], as well as the adenovirus E1B55k/E4orf6 proteins [64], assemble a complex with an E3 ligase protein to catalyze p53 ubiquitination and its subsequent degradation by the proteasome.

Here, we observed higher levels of Ubiquitin C-Terminal Hydrolase L1 (UCH-L1) in severe COVID-19 patients than in the mild group (*p* ≤ 0.05). In fact, UCHL1 is a plasma biomarker that is elevated after blood–brain barrier disruption, and has been associated with severe COVID-19 outcomes [65].

Viruses can also target host immune adaptors and signaling molecules [62]. The multiple interactions of CBL with SARS-CoV-2 proteins (nsp11, nps14, nsp13, and N) indicates that these viral proteins may recruit target cellular proteins to the up-regulated CBL protein for degradation by the proteasome (Figure 6). This mechanism decreases cellular protein stability and is correlated with enhanced viral activity. Ribosomal Protein S27a (RPS27a) interacts with Epstein–Barr virus (EBV) latent membrane protein 1 (LMP1), stabilizing LMP1 through suppression of proteasome-mediated ubiquitination and, therefore, increasing EBV proliferation [66]. This type of interaction could also benefit the SARS-CoV-2 cycle, as its proteins (S, E, Orf7b, Orf3a and M) were found to physically interact with RPS27a. Nonetheless, RPS27a was down-regulated, causing a potential lack of viral protein stabilization and consequent inhibition of viral activity (Figure 6). 

RPS27a is also involved in cell-size checkpoint regulation. It has been observed that RPS27a is needed in the HBx protein (of Hepatitis B virus) micro-environment, primarily for maintaining cell size, as there was a dramatic reduction in cell size when RPS27a gene expression was knocked down. Thus, RPS27a regulates cell proliferation [67] (Figure 6). RPS27a still has not been associated with SARS-CoV-2 infection and severity; however, its interaction with the proteins of this virus, which has been previously reported, indicates that its down-regulation may disturb SARS-CoV-2 infection, similarly to the case for Hepatitis B viral infection. It seems that the cellular environment somehow attempts to limit the virus through the down-regulation of the RPS27a protein. 

The data presented also corroborated previous studies in which a general decreased transcriptional level of genes related to protein synthesis in COVID-19 positive patients in the intensive care unit (ICU) has been observed [9]. The role of the RPS27a protein, as a part of a small ribosomal sub-unit, along with other RPs and rRNAs [68], is worth mentioning, in terms of activating cellular checkpoints such as p53 expression under stress conditions, linking ribosome biogenesis to cell cycle progression [69]. As the cell cycle is directly correlated to the translation process, all of the ribosomal down-regulated proteins found in the transcriptome of severe patients may reflect a serious dysregulation in normal cell activities.

Cytoskeleton-related genes were also unregulated. When silenced, KIF14 (Kinesin Family Member 14) interrupts cell cycle progression and induces cytokinesis failure [23] (Figure 6). Knockdown experiments have shown that KIF14 suppression inhibits cell proliferation and induces apoptosis through inactivation of AKT signaling [24]. KIF14 has been associated with cervical cancer, and a knockdown model of KIF14 impacted the cell cycle by affecting cell viability, proliferation, and migration, thus placing it as a potential therapeutic target [70]. KIF14 has not yet been associated with SARS-CoV-2 infection, but it does interact with nsp9 and nsp4 (Figure 3B). SARS-CoV-2 infection may contribute to stopping the cell cycle, so it would be able to produce more viral particles. This type of interference has been observed in picornavirus infections [71] and in infectious bronchitis virus (IBV) infection—a coronavirus that infects chickens [72]. 

Interactome, proteomic, and cellular culture studies have identified cellular proteins that interact with SARS-CoV-2 viral proteins [73]. It has been reported that SARS-CoV-2 N protein suppresses the TRIM25–DDX58 (RIG-I) interaction in cell culture experiments, inhibiting interferon response induction [74] (Figure 6). Another study has shown that the N protein regulates the innate immune response in a dose-dependent manner. At low doses, N protein reduced RIG-I ubiquitination through TRIM25 interaction, as well as reducing STAT1/STAT2 phosphorylation and nuclear translocation. Meanwhile, high doses promoted the innate immune response by enhancing the phosphorylation and nuclear translocation of STAT1 and STAT2 [75]. As we found TRIM25 to be highly expressed in severely ill patients, we hypothesized that this might be a cellular compensation against N protein blocking its activity. 

Stress granules are also greatly affected by SARS-CoV-2 infection [31,32,76,77] (Figure 6). These are membrane-less organelles that store translationally silent mRNA assembled by the cell under stress [78]. It has been shown that viral entry into the cell can interfere with stress granule (SG) formation through post-translational modifications, sequestering SG proteins, or forming ribonucleoprotein complexes with SG proteins [79,80]. 

Accumulating evidence has shown that lncRNAs can regulate the immune system, including inflammatory responses [81], and play an important role in mucosal inflammation [82]. In our work, around 10% of the DEGs were lncRNAs, and our interactome analysis identified 27 of them (Figure 5). One of those genes was TNK2-AS1, which has been shown to play a role in improving the stability of the STAT3 protein by protecting it from proteasome-mediated degradation [83] (Figure 6). Although the mRNA of STAT3 was up-regulated in our data—likely as a response of the cell to the viral infection—its protein levels may be severely impacted, as TNK2-AS1 was one of the most down-regulated genes of the network (Figure 5). Another lncRNA that has a role in the inflammatory response is AC007278.2, which represses the transcription of CCR7 by inhibiting its promoter [84] (Figure 6). Our data corroborate these findings, as CCR7 was down-regulated and AC007278.2 was up-regulated (Figure 5).

Other lncRNAs with predicted roles in the immune system that were found to be dysregulated in our analysis were PELATON, PLBD1-AS1, LINC01093, LINC01550, and NEAT1 [85,86,87,88]. NEAT1 is the core structural component of paraspeckle organelles. It acts as a transcriptional regulator for numerous genes and has previously been found to be over-expressed in severe cases of COVID-19 [89], corroborating the results of our interactome analysis (Figure S2). A recent study has analyzed the expression levels of select airway lncRNAs and found that, compared with the samples with a low viral load of SARS-CoV-2, the expression level of NEAT1 was significantly up-regulated in samples with a high viral load. In addition, when analyzed individually, the expression level of lncRNA NEAT1 was significantly correlated with the SARS-CoV-2 viral RNA level. These data suggest that NEAT1 lncRNA may play a role in the innate airway responses and may be involved in SARS-CoV-2-associated airway inflammation [82].

Considering that FBL is a 2′-O-Methyltransferase, and that it was tightly connected with lncRNAs in our interactome analysis (Figures S3–S5), one of the roles of this protein could involve the regulation of these molecules. RNA 2′-O-Methylation has the potential to impact the structure, stability, interactions, and epigenetic gene regulation of RNAs and has also been shown to play a role in human diseases [28]. The 2′-O-methyltransferase FBL plays a central role in ribosome biosynthesis, and GO enrichment analysis of the FBL interactomes identified several biological processes related to ribosomes (Tables S5–S7). As our data showed that the majority of RPs were down-regulated, as well as that the ribosome pathway was the most impacted in severe patients (Figure 3B), it is likely that FBL might play a central role in this process.

The N protein of the SARS-CoV-2 interacts with FBL (Figure 5); this interaction has already been studied in a member of the Coronavirus genus: the Infectious bronchitis virus [72]. This interaction has been implicated to play a role in delaying the cell cycle in interphase, in order to maximize the production of viral proteins [72]. These data corroborate our analysis, as several biological processes related to the cell cycle were found to be enriched when considering the proteins found in the FBL interactomes (Tables S5–S7). Hence, FBL appears to play a key role in cell cycle regulation—one of the most impaired pathways in our analysis (Figure 3B).

RNA post-transcriptional modifications stand out as a basic mechanism of transcript and protein regulation [90]. SARS-CoV-2 interferes with this process in various ways; for example, NSP1 destabilizes a certain type of mRNA to evade antiviral proteins [91]. FBL is a protein responsible for RNA modification, with multiple possible important targets being down-regulated in critically ill individuals. 

The 2′-O-methyltransferase catalyzes the formation of 2′-O-Me at the 5′-end of SARS-CoV-2 RNA, in order to impede degradation by 5′ exoribonucleases and promote uncontrolled replication and efficient translation, and to evade recognition by the host cell innate immune system through the inhibition of interferon production by immune system cells [92]. Even though FBL was down-regulated, the virus possesses its own enzyme—nsp16—to perform the 2′-O-methyltransferase reaction, allowing it to continue its replicative cycle [93].

## 4. Conclusions

The down-regulation of FBL in severe patients of COVID-19 with 50% lethality frequency was shown to play pivotal roles in impairing ribosome function and in cell cycle arrest, maximizing the transcription and translation of the SARS-CoV-2 genes while decreasing the production of host genes. Moreover, our interactome analysis indicated that, through lncRNAs, FBL is connected to down-regulated transcription-, translation-, and ribosome-related genes, highlighting the role of RNA 2′-O-methylation in the pathogenesis of SARS-CoV-2.

## 5. Materials and Methods

### 5.1. Patient Cohort and Sample Processing

Patients enrolled in this study were admitted to the University Hospital Clementino Fraga Filho (HUCFF) in Rio de Janeiro with a molecular diagnosis of positive for SARS-CoV-2, and all of them (or their representatives) signed the free and informed consent form. This study was approved by the Ethical Committee of University Hospital Clementino Fraga Filho (HUCFF), number CAAE: 31240120.0.0000.5257. Patients with COVID-19 were further categorized into hospitalized severe or mild infection, according to disease severity based on WHO guidelines (https://www.who.int/publications/i/item/clinical-management-of-covid-19 (accessed on 1 July 2020)). By matching criteria in severity scales, comorbidities, gender, and age group, 17 patients were selected for this analysis.

Between July 2020 and October 2021, the collection of 2.5–10 mL blood samples was performed, according to standard procedures, in tubes containing anticoagulants. The Buffy Coat fraction was separated by centrifugation at 1500–2000× *g* for 10–15 min at room temperature, then stored in a ratio of 1:2 of RNA at −80 °C until RNA extraction.

### 5.2. SARS-CoV-2 Detection and Quantification

In order to confirm the SARS-CoV-2 infection for all of the patients included here, the bronchial aspirate or naso-oropharyngeal swab samples were collected and processed after at least 30 min of incubation in Viral Transportation Medium (VTM). After that, for all of these samples, a RT-qPCR assay for detection and quantification of the E region of SARS-CoV-2 (Bio-Manguinhos, Rio de Janeiro, Brazil) was performed immediately after viral RNA purification. Both RNA purification and the RT-qPCR plate setup were performed by Janus 360 (PerkinElmer, Waltham, MA, USA). An in-house single-stranded RNA (ssRNA) standard curve, which was previously validated, was used to determine the SARS-CoV-2 viral load of each sample. Samples were tested in duplicate, and cycle threshold (Ct) values lower than 38.0 for the E region were considered positive.

### 5.3. Severity Biomarker Investigation

Serum levels of NfL (Neurofilament Light Chain), TAU, GFAP (Glial fibrillary acidic protein), and UCH-L1 (ubiquitin carboxyl-terminal esterase L1) were quantified using the SIMOA^®^ platform of the Quanterix SR-X instrument (Quanterix Corporation, Lexington, MA, USA), using a Neuro 4-plex A™ Kit (Quanterix Corporation, Lexington, MA, USA). Plasma samples were centrifuged (10,000× *g* for 5 min) and then diluted (1:4). All protocols were performed according to the manufacturer’s instructions. Standard calibration curves were used for quantification (0–500 pg/mL for NfL; 0–100 pg/mL for TAU; 0–1000 pg/mL for GFAP; 0–10 ng/mL for UCH-L1), and the detection limits were established by analog and digital controls. The detection limits for the four proteins were 0.136 pg/mL for NfL, 0.0298 pg/mL for TAU, 0.276 pg/mL for GFAP, and 4.03 pg/mL for UCH-L1. The calibration curves, controls, samples, paramagnetic carboxylated microspheres, and detector were incubated in 96-well plates for 30 min at 35 °C at 800 rpm. After the incubation period, the plate was washed, and Streptavidin β-galactosidase (SβG) was added before further incubation for 10 min at 35 °C and 800 rpm. After a second wash, the plate was inserted into the SR-X equipment, and reading and quantification were performed by the equipment’s specific software.

### 5.4. RNA Extraction and Library Preparation

Total RNA was extracted from Buffy Coat samples using a RiboPure™ RNA Purification Kit (Thermo Fisher Scientific, San Jose, CA, USA), according to the manufacturer’s instructions. The purity, concentration, and integrity of total RNA were measured using a NanoPhotometer spectrophotometer, Qubit RNA Assay Kit in Qubit 2.0 Fluorometer (Life Technologies, Carlsbad, CA, USA), and TapeStation (Agilent Technologies, Santa Clara, CA, USA), respectively. An average amount of 0.3 μg total RNA was used to build the libraries, using a TruSeq Stranded Total RNA Library Prep Gold Kit (Illumina, San Diego, CA, USA), according to the manufacturer’s protocol. The Illumina NextSeq platform was used to generate 75 bp paired-end reads. 

### 5.5. RNA Sequencing (RNA-seq) Analysis

The quality of the 17 transcriptome libraries was checked using FASTQC (https://www.bioinformatics.babraham.ac.uk/projects/fastqc/ (accessed on 26 August 2022)), and trimming was carried out using Trimmomatic [94]. The STAR tool [95], version 2.6, was applied to map the transcriptome libraries onto the Human genome (GRCH38.p12) from ENSEMBL databank (http://www.ensembl.org/info/data/ftp/index.html (accessed on 26 August 2022)), using the default parameters. The DESeq2 package [96] for R was used for differential gene expression analysis. Only the genes with adjusted *p*-value < 0.05 and Log_2_ fold-change > |1.0| were considered for downstream analyses.

### 5.6. Enrichment Analysis

Gene Ontology (GO) was used for the enrichment analysis of biological processes. Analysis of the transcriptome was performed using the R package GOstats [97]. The Benjamini and Hochberg (BH) adjusted *p*-value method was applied for the enrichment, with a cutoff of 0.01. The KEGG analysis was performed using the R/Bioconductor package pathview [98] using the KEGG, Biomart, and org.hs.eg.db data sets. 

### 5.7. Interactome Analysis

To produce the up- and down-regulated interaction networks, we used the data from the BioGRID database [73]. Only the 10 genes with the highest number of interactions were used for the analysis. The Cytoscape software was used for the network analysis [99]. We used the interaction information between the lncRNAs and cellular proteins extracted from the NPInter v4.0 database [100] to build a network between the lncRNAs and DEGs found in this study. Data regarding the interaction with SARS-CoV-2 viral proteins were also added, obtained from the BioGRID COVID-19 Coronavirus Curation Project. The interactome of the FBL protein was constructed using Boost Graph Library (v. 1.74.0). The weights of the nodes were calculated adding the inverse of the log_2_ Fold-Change value (1/log_2_FC). Using the Dijkstra algorithm, the shortest path connecting FBL to each of the DEGs was calculated. Cytoscape was used to visualize all interaction networks and to calculate centrality scores. 

### 5.8. Statistical Analysis

For the statistical analysis, a non-parametric *t*-test was applied for the comparison between the two unpaired groups; for the U-test, the Mann–Whitney approach was applied. These statistical tests were performed using the GraphPad Prism v.9 software (GraphPad—San Diego, CA, USA).

## Figures and Tables

**Table 1:** Laboratory and clinical features. Comorbidity, viral load, and serum biomarker levels of light chain neurofilament, glial fibrillary acid protein, ubiquitin, and TAU total protein among COVID-19 severe and mild patients.

Laboratory and Clinical Features	Severe (*n* = 12)	Mild (*n* = 5)	*p*-Value
SARS-Cov-2 E viral load (median) *	6620	13.8	0.0059
Hospitalization (in days; median)	14	NA	-
Hypertension	(10) 83.3 %	(1) 20.0 %	-
Diabetes	(9) 75.0	0	-
Obesity	0	0	-
Dyslipidemia	0	0	-
Pneumonia	(12) 100%	0	-
Oxygen supplementation	(12) 100%	0	-
SOFA index (median)	6	NA	-
Neurofilament plasma level (median)	91.80 pg/mL	14.52 pg/mL	0.0275
Glial fibrillary acid protein plasma level (median)	346.0 pg/mL	143.9 pg/mL	0.0517
Ubiquitin carboxy-terminal hydroxylase L1 plasma level (median)	100.1 pg/mL	38.83 pg/mL	0.0133
TAU total protein plasma level (median)	6.668 pg/mL	2.599 pg/mL	0.0133
Lethality	(6) 50%	0	-

Abbreviations: SOFA, Sequential Organ Failure Assessment Score; M, male; F, female; Y, years; NA, not applicable; * PFU/Ml, plaque-forming units.

## Data Availability

The datasets used in the study are available online via the Gene Expression Omnibus database under accession number PRJNA870930.

## Supplementary Materials

The supporting information can be downloaded at: https://www.mdpi.com/article/10.3390/ijms232113588/s1.
