# Supplementary material for: Buffy Coat Transcriptomic Analysis Reveals Alterations in Host Cell Protein Synthesis and Cell Cycle in Severe COVID-19 Patients
Source: Int J Mol Sci. 2022 Nov 5;23(21):13588. doi: 10.3390/ijms232113588 (PMC9659271; doi:10.3390/ijms232113588)
Supplement: Supplementary file 1 [file ijms-23-13588-s001.zip › Supplementary Figures.pdf]

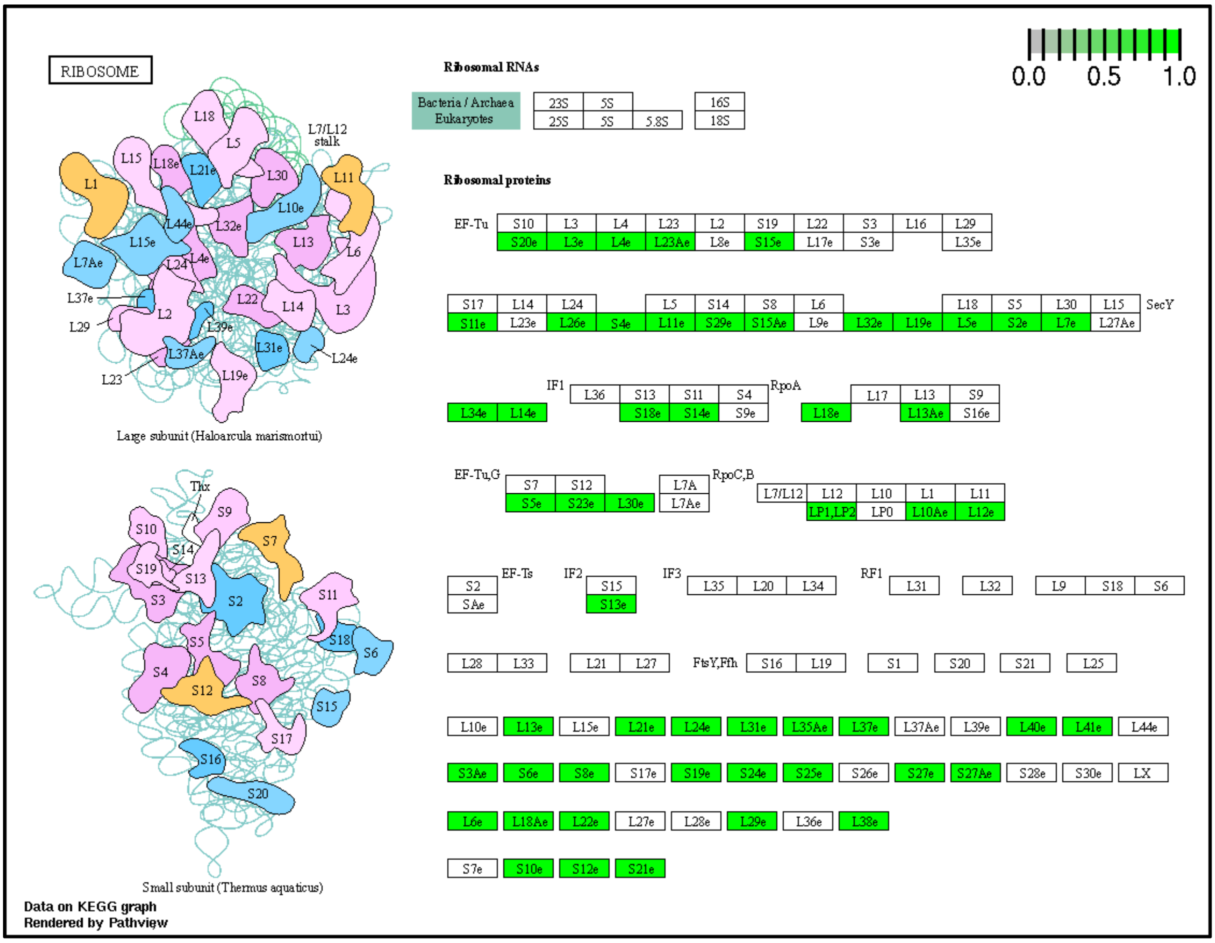

Figure S1. KEGG ribosome pathway. In green are the downregulated genes identified in the RNA-seq anaysis.

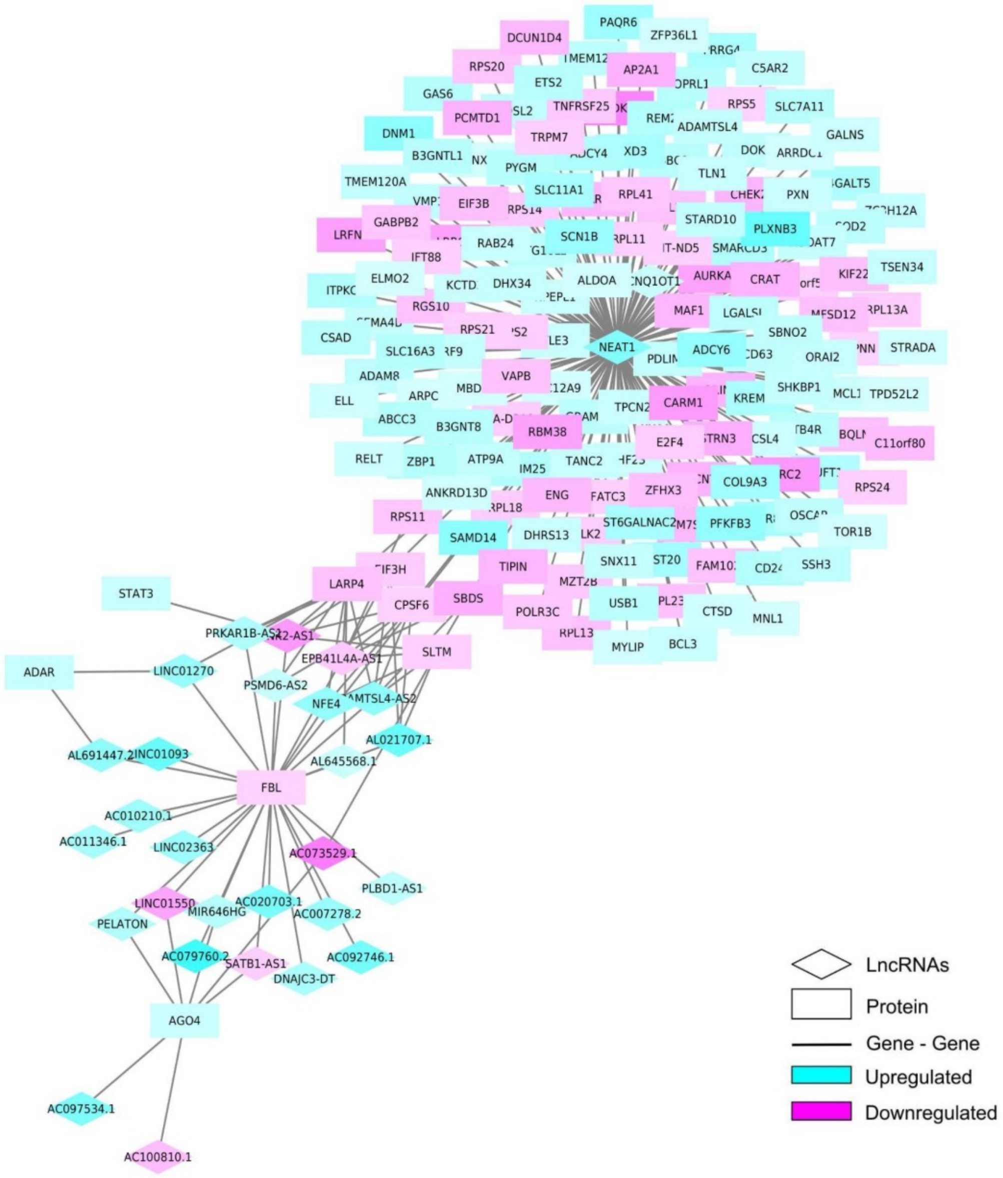

Figure S2. Protein-coding lncRNAs interaction network. This network was constructed with all differentially expressed genes that have a connection with differentially expressed protein-coding genes in the RNA-seq analysis.

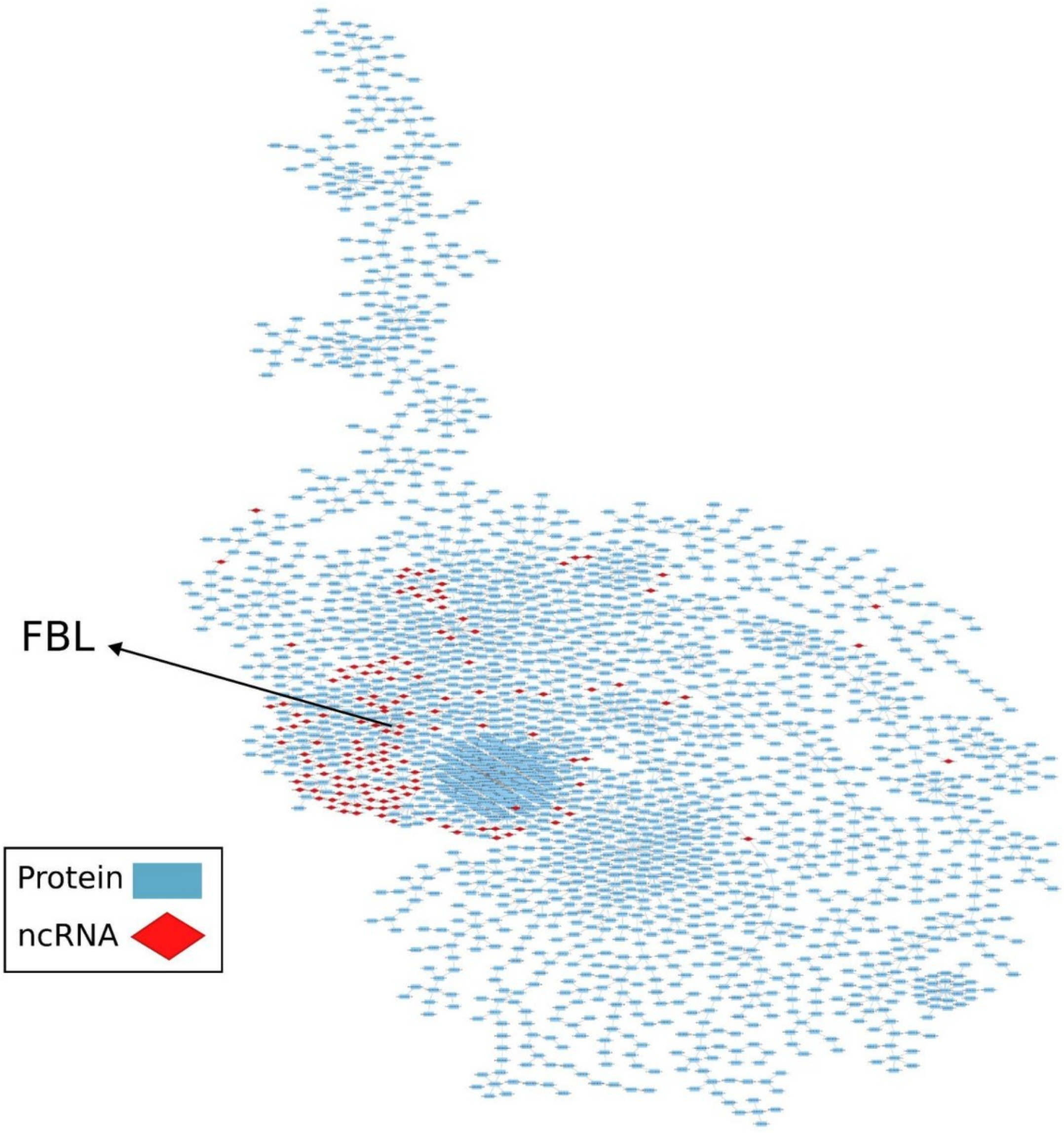

Figure S3. FBL Interactive network all DEGs. Interactive network of the FBL protein connected to all DEGs using the Dijkstra algorithm.

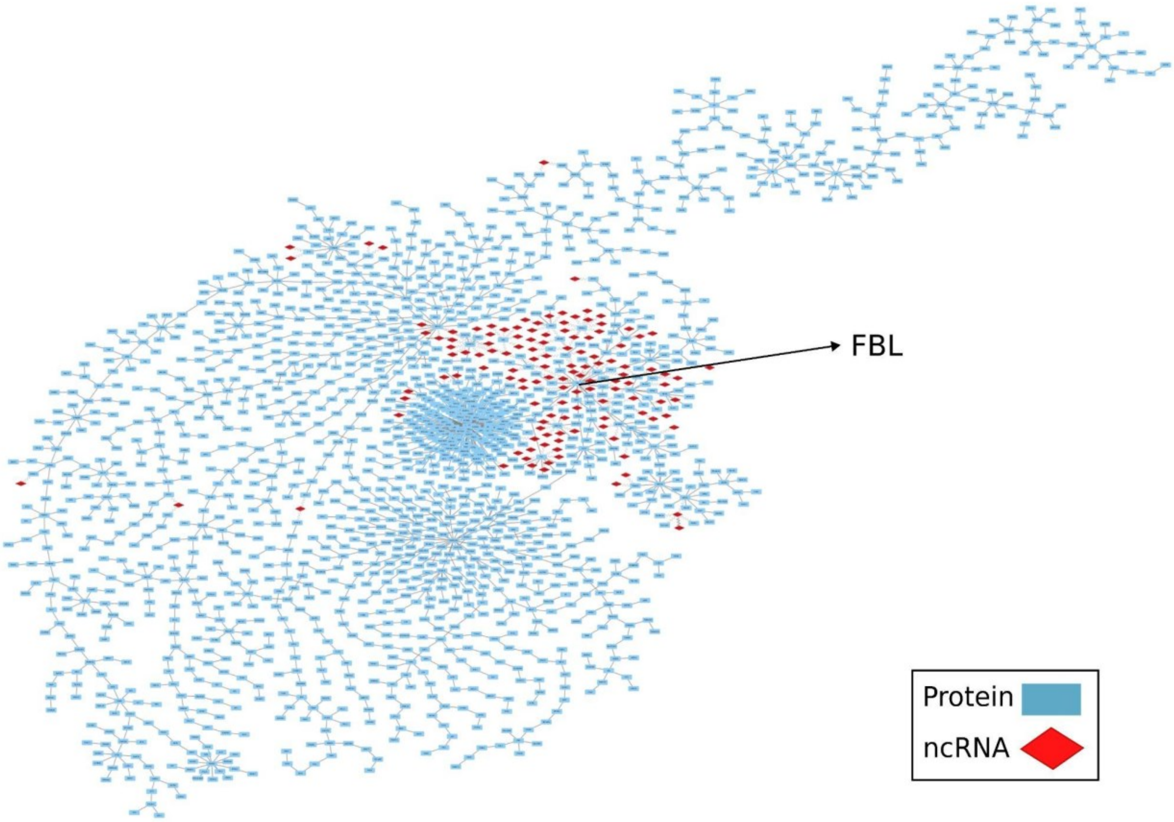

Figure S4. FBL Interactive network upregulated DEGs. Interactive network of the FBL protein connected to all upregulated DEGs using the Dijkstra algorithm.

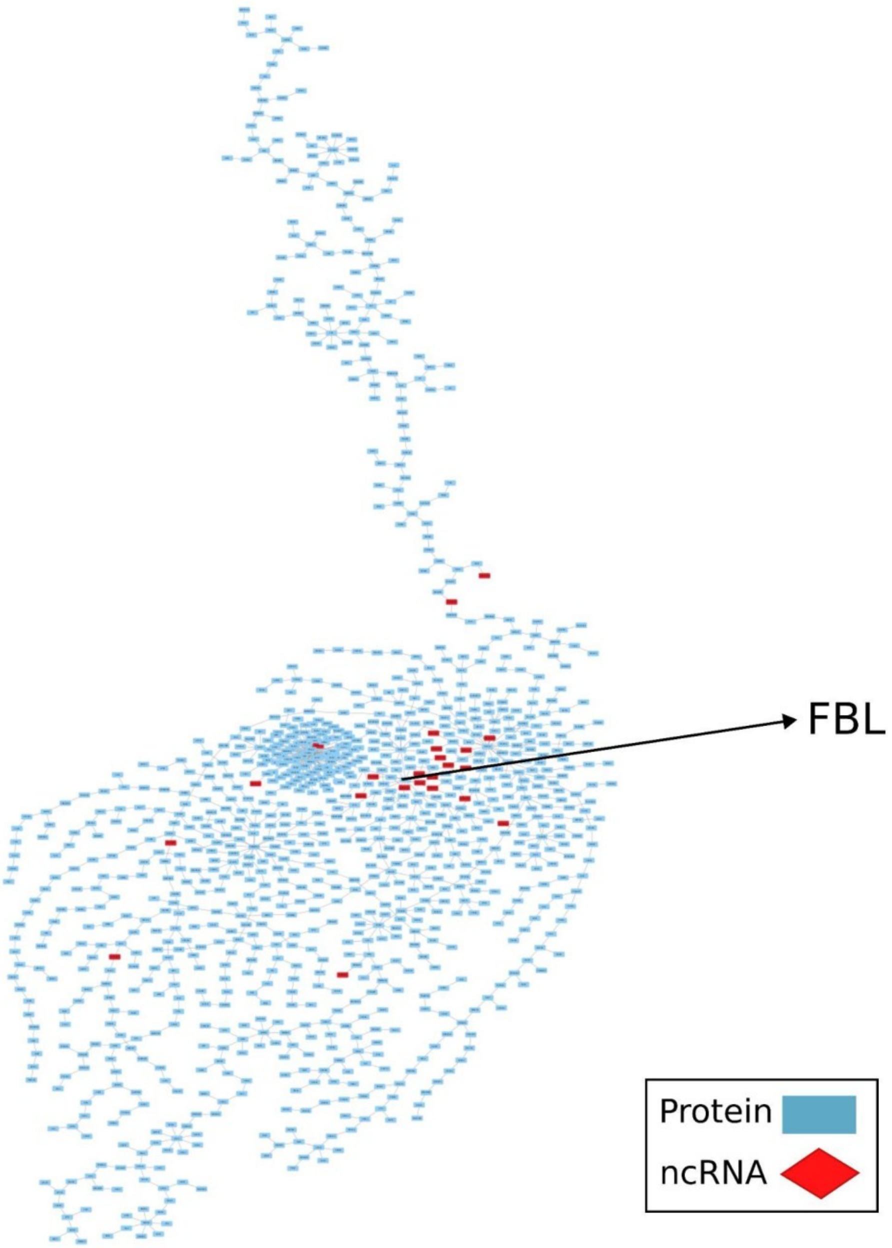

Figure S5. FBL Interactive network downregulated DEGs. Interactive network of the FBL protein connected to all downregulated DEGs using the Dijkstra algorithm.
